# Effect of folic acid combined with pravastatin on arteriosclerosis in elderly hypertensive patients with lacunar infarction

**DOI:** 10.1097/MD.0000000000026540

**Published:** 2021-07-16

**Authors:** Chunxia Li, Xingpeng Bu, Yaru Liu

**Affiliations:** Shanxi Bethune Hospital, Shanxi Academy of Medical Sciences, Tongji Shanxi Hospital, Third Hospital of Shanxi Medical University, Taiyuan, China.

**Keywords:** atherosclerosis, elderly hypertension, folic acid, lacunar infarction, pravastatin

## Abstract

This study aimed to assess the effect of folic acid combined with pravastatin on atherosclerosis-related indexes in elderly patients with hypertension complicated with lacunar cerebral infarction.

A total of 134 elderly hypertensive patients with lacunar cerebral infarction were randomly divided into 3 groups using the random number table method. Group A, the folic acid group, had 45 cases and received low-dose folic acid (0.8 mg/d) treatment on the basis of antihypertensive treatment. Group B, the pravastatin group, had 45 cases and received pravastatin (20 mg/d) treatment on the basis of antihypertensive treatment. Group C, the folic acid combined with the pravastatin group, had 44 cases. Members of this group received pravastatin (20 mg/d) and low-dose folic acid (0.8 mg/d) based on antihypertensive treatment. Levels of folic acid, homocysteine (Hcy), tumor necrosis factor alpha (TNF-a), matrix metallopeptidase 9 (MMP-9), cholesterol (TC), and low-density lipoprotein cholesterol (LDL-C) were measured by ELISA before treatment in all 3 groups. Carotid intima-media thickness (IMT) was measured using ultrasound, and systolic and diastolic blood pressure were measured with a mercury column. After 8 weeks of treatment, the levels of folic acid, Hcy, TNF-a, MMP-9, TC, LDL-C, and systolic and diastolic blood pressure were compared among the 3 groups. IMT levels were measured at 12 weeks of treatment.

After 8 weeks of treatment, compared with group B, patients in groups A and C had folic acid levels significantly higher than baseline levels, with significantly lower Hcy levels (both *P* < .05). Patients in group C presented significantly decreased TNF-a, MMP-9, TC, and LDL-C levels and systolic and diastolic blood pressure after 8 weeks of treatment, compared with those in groups A and B (both *P* < .05). These patients also showed significantly decreased IMT levels compared with those in the other groups (*P* < .05).

Low-dose folic acid combined with pravastatin in elderly patients with lacunar cerebral infarction can reduce the level of homocysteine, improve the degree of carotid atherosclerosis, protect vascular endothelium, and reduce blood lipids and blood pressure, presenting better benefits than pravastatin alone.

## 1 Introduction

Lacunar cerebral infarction is caused by cerebral ischemia in the deep brain stem or cerebral hemisphere, a small artery occlusion caused by a glassy or small embolism of arterioles. It is an ischemic stroke. It affects the sensory and motor systems and affects the brain's cognitive function, causing cognitive dysfunction in patients. The clinical manifestations of lacunar infarction are mild; as such, they are not valued.^[1]^

At present, small vessel atherosclerosis is the pathophysiological basis of lacunar cerebral infarction. Homocysteine (Hcy) is an important intermediate in the metabolism of methionine and cysteine in the human body. Its elevation can affect lipid metabolism and the autooxidation process, damage vascular endothelial cells, and promote the progression of atherosclerosis. Hcy has been determined as an independent risk factor for cardiovascular and cerebrovascular diseases; reduction of its levels contributes to the prevention and treatment of cerebrovascular diseases.^[2]^

In elderly patients, folic acid absorption disorders are often caused by decreased food intake, reduced digestive function, and decreased absorption and utilization of nutrients. These lead to hyperhomocysteinemia, which increases the risk of stroke. Studies have confirmed that exogenous folic acid can reduce plasma Hcy levels and reduce the risk of hyperhomocysteinemia. It has a positive effect on the prevention and treatment of vascular diseases,^[3]^ and it protects against hypertension, atherosclerosis, and vascular endothelium.^[4]^ Many studies have confirmed that folic acid has a stabilizing effect on arterial plaque, and the supplementation of low-dose folic acid is also beneficial to lower total cholesterol and low-density lipoprotein (LDL).^[5]^

A meta-analysis of 369,746 people observed in 11 prospective cohort studies showed that increasing folic acid intake can reduce the risk of coronary heart disease.^[6]^ Another study showed that folic acid can reduce the risk of in-stent restenosis after coronary intervention.^[7]^ These studies have shown that folic acid has a protective effect on the stability of arterial plaque, and its mechanism may be closely related to its anti-inflammatory effect. Folic acid can reduce the body's pro-inflammatory cytokine monocyte chemoattractant protein-1 level.^[8,9]^ Monocyte chemoattractant protein-1 plays an important role in the formation and development of unstable plaques and can reduce the stability of plaques by promoting the aggregation of macrophages, the release of inflammatory factors, and the formation of foam cells.^[10]^ Therefore, folic acid may have a more positive effect on improving arteriosclerosis, promoting plaque stability, and lowering cholesterol. Tumor necrosis factor alpha (TNF-a) is currently recognized as a vascular endothelial inflammatory factor, a crucial factor in promoting vascular endothelial injury that leads to atherosclerosis. Matrix metallopeptidase 9 (MMP-9) plays a vital role in atherosclerosis and plaque formation. Therefore, increased folic acid intake in elderly hypertension patients with lacunar infarction not only reduces homocysteine but may also synergistically reduce LDL and promote plaque stabilization.

In addition to lowering lipids, statins can inhibit the vascular endothelial inflammatory reaction and delay the progression of arteriosclerosis, which has good application value.^[11]^ Statins are the basic drug treatment for elderly patients with hypertension and lacunar infarction. Although statins are drugs that have been proven to stabilize plaque and reduce low-density lipoproteins, in clinical practice, the effect of single use of statins is often not favorable—patients’ LDL levels do not meet the standards, and the risk of cerebral infarction occurs again during medication. This phenomenon is more common in elderly patients.

The elderly often suffers from a variety of chronic diseases and need to take a variety of medications. They also experience liver and kidney dysfunction and pharmacokinetic changes. To control the incidence of cardiovascular and cerebrovascular diseases among this patient population, there is a clinical need for increased statins to control low-density lipoproteins, which may lead to risks of liver damage, myolysis, renal insufficiency, etc. As a kind of vitamin, folic acid increases the intake without risk of liver and kidney dysfunction. There have been no studies in the literature addressing the potential effect of low-dose folic acid in elderly patients with hypertension and lacunar infarction.

Therefore, this study investigated whether low-dose folic acid has a positive effect on vascular endothelial inflammatory factors, cholesterol, LDL, plaque stabilization, and blood pressure reduction in elderly patients with hypertension and lacunar infarction. We also assessed the effect of folic acid combined with pravastatin as a potential option for patients with lacunar infarction. The folic acid used in this study is the same as that used by pregnant women, which has no toxic side effects.

Folic acid is a B vitamin. If a woman has enough folic acid in her body before and during early pregnancy, it can help prevent neural tube defects. Women need folic acid every day. In 1992, the U.S. Public Health Service recommended that all women of childbearing age capable of becoming pregnant consume folic acid daily to prevent neural tube defects. By 1998 in the United States, folic acid was added to all cereal grain products labeled as enriched, like breakfast cereals and corn grits. This process is called folic acid fortification. In the United States, fortification led to a 35% decrease in the rate of neural tube defects.^[12–15]^ Fortifying foods with folic acid is an effective way to increase a woman's intake of folic acid without changing her dietary habits.

To the best of our knowledge, this report evaluated the combined use of folic acid and pravastatin to determine its effectiveness in reducing the incidence of stroke without increasing the risk of liver and kidney dysfunction.

## 2 Materials and methods

### 2.1 General data

We selected 134 patients with senile hypertension complicated with lacunar infarction who were admitted to our hospital from December 2014 to December 2016 as subjects. All patients met the diagnostic criteria for senile hypertension^[16]^ and the diagnosis of lacunar infarction.^[17]^ Standard and diagnostic criteria for dyslipidemia were used.^[18]^ Patients were aged 60 to 80 years, had no previous history of cerebral infarction, and sex was not limited. The Hcy level was ≥10.0 μmol/L. All 3 groups had not received statins, folic acid, or B vitamins before 8 weeks to ensure the reliability of the test data. Patients had normal cognitive function, good compliance, informed knowledge about the study, and signed informed consent forms. We excluded acute stroke, disturbance of consciousness, intracranial, or systemic infection; patients with malignant tumors, autoimmune diseases; coronary heart disease, secondary hypertension, type 2 diabetes; patients with congestive heart failure; brain patients with trauma, post-epileptic state, poisoning, etc; severe liver or kidney, coagulopathy; history of trauma or surgery in the past 3 months; peripheral arterial occlusive disease; alcohol or drug dependence; those who are allergic to the research drugs; cognitive dysfunction or mental illness; or patients who were to be included in other clinical research. The study was approved by the ethics committee of Shanxi Bethune Hospital.

Diagnosis of hypertension in the elderly: age ≥65 years old, blood pressure continuous or >3 times non-same day sitting systolic blood pressure ≥140 mmHg and/or diastolic blood pressure ≥90 mmHg. The diagnostic criteria for lacunar infarction were as follows: the onset of hypertension is mostly caused by arteriosclerosis, which is acute or subacute; in most cases no disturbance of consciousness occurs; computed tomography (CT) or magnetic resonance imaging (MRI) should be performed to confirm the diagnosis; and the clinical manifestations were not serious. The most common ones were pure sensory stroke, pure motor hemiplegia, ataxia hemiplegia, dysarthria hand clumsy syndrome, or sensorimotor stroke. Low-density lipoprotein cholesterol (LDL-C) <1.8 mmol/L and TC <3.1 mmol/L should be set as the target values of blood lipid control in elderly patients with hypertensive lacunar cerebral infarction, above this level is dyslipidemia.

Before the grouping, each study subject was given 2.5 to 5 mg of amlodipine orally to maintain blood pressure below 140/90 mmHg. Due to individual differences, the amounts of amlodipine administered to each subject were different to maintain no difference in blood pressure before the study.

Then, patients were divided into 3 groups A, B, and C using the random number table method, and all 3 patients continued to receive folic acid, pravastatin, or folic acid and pravastatin treatment on an oral basis of a certain amount of amlodipine until the end of observation.

There were 45 cases in group A. The group included 23 men and 22 women, aged 60 to 77 (68.62 ± 5.14) years old. Among the participants, 10 had a personal history of long-term smoking, and 3 had a history of long-term drinking. There were 45 cases in group B. The group included 22 men and 23 women, aged 60 to 78 (69.02 ± 5.37) years old. Among the participants, 9 had a history of long-term smoking and 5 had a history of long-term drinking. In group C, there were 44 cases. The group included 23 men and 21 women, aged 60 to 80 (70.01 ± 5.22) years old. There were 8 participants with a history of long-term smoking and 4 participants with a history of long-term drinking. There were no significant differences in sex, age, and personal history (clinically, personal history refers to the history of smoking and drinking) among 3 groups (*P* > .05). Long-term smoking refers to smoking for >5 years. Long-term drinking refers to drinking liquor 2 to 3 times a week for at least 5 years.

### 2.2 Treatment

All 3 groups of patients were given amlodipine 2.5 to 5 mg/d, maintaining blood pressure below 140/90 mmHg, while being given nutritional nerve, blood pressure, blood sugar, anticoagulation, antiplatelet agglutination, and other treatments. These treatments are standardized treatments for patients with lacunar infarction. Group A was given a small dose (0.8 mg) of folic acid (Beijing Silean Pharmaceutical Co., Ltd., Jiangxi Pharmaceutical Co., Ltd., approval number: National Pharmaceutical Standard H1097007936020872, specification: 0.4 mg) each day, taken as an oral treatment after breakfast. Group B was given 20 mg of pravastatin (First Sankyo Pharmaceutical [Shanghai] Co., Ltd., approval number: National Drug Standard H200332150150, specification: 40 mg) each day, taken before bedtime. Group C was treated with a low dose of folic acid (0.8 mg/d, after breakfast) and pravastatin (20 mg/d, before bedtime). All groups were treated for 12 weeks. During the study period, all 3 groups were given a uniform standard of diet and daily activity patterns.

### 2.3 Observation index

Before treatment, fasting venous blood was taken from the participants. The plasma levels of folic acid (μg/L), Hcy (μmol/L), TNF-a (pg/mL), MMP-9 (ng/mL), cholesterol test (TC) (mmol/L), and LDL-C (mmol/L) were determined using enzyme-linked immunosorbent assay. The level of intima-media thickness (IMT) was measured by ultrasound, and the levels of systolic and diastolic blood pressure were measured by mercury column. Fasting venous blood was taken from the 3 participant groups after 8 weeks of treatment. Plasma folate, Hcy, TNF-a, MMP-9, TC, and LDL-C were measured using enzyme-linked immunosorbent assay. The systolic and diastolic blood pressures of the 3 groups were measured 8 weeks after treatment. IMT level was detected by ultrasound Doppler at 12 weeks of treatment.

Blood pressure readings were collected by 3 health care providers following the blood pressure measurement specifications stipulated in the hypertension guidelines. During the study period, blood pressure data were measured by the same mercury column blood pressure meter, and 3 providers shared one-third of each group of blood pressure measurements. Similarly, another 3 providers followed the operating practice process and used the same ultrasound instrument to measure the carotid intima-media thickness in one-third subgroup of each group of patients.

### 2.4 Statistical methods

Statistical data processing was performed using the SPSS 23.0 software package (SPSS inc.). The measurement data were expressed as mean ± standard deviation. The comparison between groups was analyzed using analysis of variance. The comparison within the group was done using repeated measurement data, and the data of counting were analyzed by F-test. *P* < .05 was considered statistically significant.

## 3 Results

There was no significant difference in the levels of folic acid, Hcy, TNF-a, MMP-9, TC, and LDL-C among 3 groups before treatment (*P* > .05; Table 1). These measurements are shown in Table 2. After 8 weeks of treatment, the levels of folic acid were significantly higher in groups A and C than in group B (*P* < .05); Hcy was significantly decreased, and the difference was statistically significant (*P* < .05). Compared with groups A and B, TNF-a, MMP-9, TC, and LDL-C were significantly decreased in group C, and the difference was statistically significant (*P* < .05).

**Table 1:** Comparison of levels of folic acid (μg/L), Hcy (μmol/L), TNF-a (pg/mL), MMP-9 (ng/mL), TC (mmol/L), and LDL-C (mmol/L) in the 3 groups before treatment (mean ± SE).

Group	Number of cases	Folic acid	Hcy	TNF-a	MMP-9	TC	LDL-C
A group	45	7.53 ± 5.31	17.53 ± 6.14	27.26 ± 3.15	2.08 ± 0.57	4.57 ± 1.30	2.53 ± 0.69
B group	45	7.47 ± 5.21	17.74 ± 5.37	27.17 ± 3.00	2.05 ± 0.53	4.52 ± 1.27	2.51 ± 0.63
C group	44	7.49 ± 5.26	17.47 ± 5.55	27.13 ± 3.12	2.07 ± 0.54	4.56 ± 1.28	2.52 ± 0.65
F value		1.142	2.235	0.227	1.419	0.321	0.599
*P* value		.322	.111	.797	.246	.726	.551

Hcy = homocysteine, LDL-C = low-density lipoprotein cholesterol, MMP-9 = matrix metallopeptidase 9, TC = cholesterol test, TNF-a = tumor necrosis factor alpha.

**Table 2:** Comparison of levels of folic acid (μg/L), Hcy (μmol/L), TNF-a (pg/mL), MMP-9 (ng/mL), TC (mmol/L), and LDL-C (mmol/L) in 3 groups after 8 weeks treatment (mean ± SE).

Group	Number of cases	Folic acid	Hcy	TNF-a	MMP-9	TC	LDL-C
A group	45	12.53 ± 5.31	8.53 ± 4.37	20.15 ± 3.15	100.50 ± 10.94	4.00 ± 1.21	2.21 ± 0.69
B group	45	7.47 ± 5.21	11.74 ± 5.25	19.17 ± 4.50	99.43 ± 11.01	3.88 ± 1.35	2.199 ± 0.63
C group	44	13.49 ± 5.26	7.47 ± 3.7	15.13 ± 3.12	79.48 ± 9.87	2.05 ± 1.23	1.86 ± 0.65
F value		39.01	131.5	252.4	213.6	11.58	8.658
*P* value		4.88e–14^∗∗∗^	<2e–16 ^∗∗∗^	<2e–16 ^∗∗∗^	<2e–16 ^∗∗∗^	2.33e–05 ^∗∗∗^	.000293 ^∗∗∗^
F (A and B)		65.06	23.58	2 .175	0.112	0.431	0.768
*P* value		3.4e–12 ^∗∗∗^	5.16e–06 ^∗∗∗^	0.144	0.738	0.513	.383
F (C and A)		1.215	9.079	256.8	316.5	164.9	34.06
*P*		.273	.00338 ^∗∗^	<2e–16 ^∗∗∗^	<2e–16 ^∗∗∗^	<2e–16 ^∗∗∗^	8.76e–08 ^∗∗∗^
F (C and B)		96.8	50.26	142.8	326	143.4	26.55
*P*		7.7e–16 ^∗∗∗^	3.21e–10 ^∗∗∗^	<2e–16 ^∗∗∗^	<2e–16 ^∗∗∗^	<2e–16 ^∗∗∗^	1.56e–06 ^∗∗∗^

Hcy = homocysteine, LDL-C = low-density lipoprotein cholesterol, MMP-9 = matrix metallopeptidase 9, TC = cholesterol test, TNF-a = tumor necrosis factor alpha. ^∗^.05.

∗∗ .01.

∗∗∗ .001.

Three groups of systolic and diastolic blood pressure measurements were compared. The results are shown in Table 3. Before treatment, there were no significant differences in systolic and diastolic blood pressure among 3 groups (*P* > .05). After 8 weeks of treatment, the systolic and diastolic blood pressure of group C was lower compared with groups A and B. Both systolic and diastolic blood pressure decreased, and the difference between baseline and after treatment values was statistically significant (*P* < .05).

**Table 3:** Comparison of blood pressure levels in 3 groups (mean ± SE, mmHg).

		Systolic blood pressure		Diastolic blood pressure	
Group	Number of cases	Before treatment	After treatment 8 weeks	Before treatment	After treatment 8 weeks
A group	45	140.7 ± 12.7	136.7 ± 5.7	87.6 ± 10.2	78.2 ± 8.1
B group	45	138.2 ± 14.6	133.9 ± 6.7	88.3 ± 9.0	79.0 ± 8.6
C group	44	139.4 ± 13.1	129.2 ± 5.8	89.1 ± 9.9	70.2 ± 7.1
F (A , B and C)		1.42	7.115	1.557	61.19
P		0.25	0.00116^∗∗^	0.215	<2e–16^∗∗∗^
F (C and A)		0.079	128.9	1.873	92.06
*P*		.779	<2e–16^∗∗∗^	.175	2.44e–15^∗∗∗^
F (C and B)		0.095	64.03	2.835	106.6
*P*		0.759	4.59e–12^∗∗∗^	0.0958	<2e–16^∗∗∗^

Table 4 shows the IMT levels of the 3 participant groups. There was no significant difference in IMT level among 3 groups before treatment (*P* > .05). After 12 weeks of treatment, compared with groups A and B, the IMT level of group C was significantly lower (*P* < .05).

**Table 4:** Comparison of 3 groups of treatments at different time IMT (mean ± SE, mmHg).

Group	Number of cases	Before treatment	After treatment 12 weeks
A group	45	1.63 ± 0.11	1.16 ± 0.14
B group	45	1.67 ± 0.12	1.12 ± 0.16
C group	44	1.65 ± 0.14	1.01 ± 0.20
F (A, B and C)	1.521	22.49	
*P*	.222	3.94e–09^∗∗∗^	
F (C and A)	0.01	44.82	
*P*	.922	1.95e–09^∗∗∗^	
F (C and B)	2.67	16.59	
*P*	.106	.000101^∗∗∗^	

^∗^.05. ^∗∗^.01.

∗∗∗ .001.

An evaluation of adverse reactions revealed that none of the 3 groups had allergic reactions, liver, and kidney function damage, increased creatine kinase, or acute cardiovascular and cerebrovascular events. The main manifestations were gastrointestinal symptoms. In group A, there were 2 cases of loss of appetite, 2 cases of nausea, and 3 cases of abdominal distension, with a total incidence rate of 15.56%. In group B, there were 3 cases of loss of appetite, 1 case of nausea, and 4 cases of abdominal distension, with a total incidence rate of 17.78%. In group C, there were 2 cases of loss of appetite, 2 cases of nausea, and 3 cases of abdominal distension, with a total incidence rate of 15.9%. There was no significant difference in the incidence of adverse reactions among the 3 groups (X^2^ = 0.093, *P* > .05). The loss of appetite was a short-term symptom, which appeared early and was gradually improved without affecting the health of patients.

## 4 Discussion

As society ages, the elderly is the main population susceptible to cerebrovascular diseases and death. Due to atherosclerosis of the arterial vasculature in elderly patients, vascular compliance is reduced, and it is easy to have unstable blood pressure and vascular endothelial dysfunction. The incidence of cardiovascular and cerebrovascular complications (e.g., hypertension, cerebral infarction, coronary heart disease, and carotid atherosclerosis increases), and their early diagnosis and treatment can greatly control patients’ conditions.

Lacunar infarction is a common type of cerebral infarction in the elderly. It is often secondary to senile hypertension. It is ischemic cerebral infarction caused by occlusion of deep perforating branches in the cerebral hemisphere or brain stem. Mild, clinical symptoms of lacunar infarction are not typical, only manifesting as cortical dysfunction or emotional changes. As a result, they are easy to ignore and eventually lead to the formation of multiple areas or even large areas of cerebral infarction, resulting in cognitive decline or disability and death.^[19]^

The pathophysiological basis of lacunar infarction is atherosclerosis. The vascular wall gradually calcifies, with increased stiffness and decreased compliance. An abnormal activation of the inflammatory response leads to local infiltration of macrophages and phagocytosis of arterial intima deposits. Lipids become foam cells, forming the earliest lipid streaks of atherosclerotic lesions. Hemodynamic changes, including damaged arterial intima endothelial cells and platelet adhesion and aggregation and activation of platelet activating factor, are observed. On the intima, thrombus is formed, and a large number of inflammatory cytokines are released. These cytokines enter the blood vessel wall and promote smooth muscle cell proliferation. With the accumulation and migration of a large number of inflammatory cells and the proliferation of smooth muscle cells, inflammatory cells continue to release various cytokines and chemokines, ultimately leading to the formation of atherosclerotic plaques. Therefore, the inflammatory response and smooth muscle cell proliferation play a vital role in the formation and progression of atherosclerosis.^[20,21]^

Hcy is a sulfur-containing amino acid, an important intermediate in the metabolism of methionine and cysteine in the human body. Hcy is a risk factor for atherosclerosis, hypertension, and thromboembolic diseases. The incidence of cardiovascular and cerebrovascular diseases is increasing in China. About 75% of elderly hypertensive patients in China show an increase in Hcy. This increase can cause poor blood pressure control and may lead to vascular endothelial dysfunction in the body, resulting in atherosclerosis. Hcy promotes atherosclerosis by initiating a 3-step mechanism. First, it triggers the release of various cytokines and active cell substances, leading to damage of the arterial wall.^[22]^ As observed in animal studies, Hcy next damages the vascular endothelium by mediating oxidative stress, promoting smooth muscle cell proliferation, and increasing foam cell production promotes atherosclerosis.^[23]^ Lastly, it promotes platelet aggregation, thrombosis, and the deposition of fibrin and thrombus in arterial blood vessels.^[24]^

Older hypertensive patients with increased Hcy may have a 5-fold higher risk of atherosclerosis than persons with uncomplicated hypertension.^[25]^ Similarly, studies have shown that hyperhomocysteinemia is associated with the occurrence of lacunar infarction, which involves Hcy in its development process.^[26]^ Therefore, the reduction of Hcy with drugs based on conventional antihypertensive, lipid-lowering, anti-platelet therapy, etc, can help control patients with hypertension and lacunar infarction.

TNF-a is an important member of the tumor necrosis factor family. It is a pro-inflammatory factor produced by monocytes and macrophages, and acts on vascular endothelial cells, damaging endothelial cells or causing vascular dysfunction and damage. Thrombosis plays a crucial role in the development of arteriosclerosis.^[27]^

Previous studies suggest that MMP-9 is involved in the formation of plaque fibrous caps, which can lead to plaque instability and are closely related to atherosclerosis, the severity of cerebral infarction, cognitive decline, and the prognosis of cerebrovascular disease. Studies have confirmed that MMP-9 gene polymorphism is associated with the increased prevalence of cerebral infarction.^[28]^

Pravastatin is a third-generation statin lipid-lowering drug. It can inhibit inflammatory cytokines, reduce the expression of leukocytes and endothelial cell adhesion molecules, and has anti-inflammatory effects. It improves vascular endothelial function, reduces plaque tension, improves the stability of atherosclerotic plaque, improves platelet function, reduces the formation of thrombosis, and prevents the formation of atherosclerosis through various mechanisms. The drug was suitable for our study because of its water solubility. Pravastatin metabolism is independent of the liver's drug metabolism, and its metabolites have no drug activity. Therefore, its side effects on the liver are relatively small. Moreover, elderly patients often need to take many drugs at the same time. Drug metabolism occurs through the cell P450 system, and because pravastatin is not metabolized by this system, the drug interaction is lower, making it safer for elderly patients.

Folic acid is a water-soluble B vitamin. It is involved in the body's synthesis and metabolism under the catalysis of methylenetetrahydrofolate reductase. When the body is deficient in folic acid or methylenetetrahydrofolate reductase, it will cause abnormal Hcy metabolism. The accumulation of Hcy in the body damages the endothelial cells of the wall, leading to atherosclerosis and causing structural and functional disorders along the vessel wall.^[29]^ Oral supplementation with folic acid is a safe and effective method for reducing Hcy. The combined use of antihypertensive drugs and folic acid in patients with hypertension and elevated levels of Hcy can improve blood pressure control and reduce Hcy levels.^[30,31]^ Pushpakumar et al^[32]^ studied the protective effects of folic acid in hypertension and confirmed that folic acid supplementation has a protective effect on cardiovascular events in hypertension. Other studies, including experiments in rats, have shown that low-dose folic acid as a supplement can significantly reduce their Hcy, TC, and low-density lipoprotein concentrations and lower the incidence of atherosclerosis, suggesting that low-dose folic acid can be used as an atheroma and primary prevention of sclerosis in subjects with risk factors for atherosclerosis.^[33]^ Related studies have also confirmed that the addition of an appropriate amount of folic acid can reduce the occurrence of aortic aneurysm^[4]^ and inhibit the formation of venous thrombosis^[34]^ while protecting against the occurrence of aortic dissection.^[35]^

Current research suggests that the anti-atherosclerosis effect of folic acid may be related to its anti-inflammatory effects, but the specific mechanism is still unclear, and folic acid has rarely been reported in the study of atherosclerosis in elderly hypertensive patients with lacunar infarction. This study investigated the effect of 0.8 mg/d folic acid combined with pravastatin 20 mg/d on arteriosclerosis to assess the possible beneficial effects in elderly patients with hypertension and lacunar infarction. The impact of diet and activities was minimized by ensuring the same healthy diet and healthy activity patterns among the 3 groups. There is no statistical difference among the 3 groups before the experiment.

Compared with folic acid (group A) or pravastatin (group B) alone, patients administered folic acid combined with pravastatin (group C) had significantly low Hcy, TNF-a, MMP-9, and blood lipid levels, and their carotid intima-media thickness was also significantly improved after 12 weeks of treatment. Systolic and diastolic blood pressure were also significantly reduced, indicating that folic acid combined with pravastatin has a synergistic anti-inflammatory effect and improved arteriosclerosis. Our findings also showed that folic acid reduced Hcy levels after 8 weeks of treatment, demonstrating that supplementation with small doses of folic acid can achieve cardiovascular benefits. Folic acid alone can reduce Hcy, TNF-a, and MMP-9 to improve vascular endothelial function, reduce inflammation, lower lipid levels, and blood pressure, and result in other beneficial effects.

Blood pressure fluctuations are present in elderly hypertension patients. To ensure that the blood pressure was kept in a relatively safe range during the study and that there was no significant difference in blood pressure among 3 groups, patients were given the same medicines to maintain their blood pressure below 140/90 mmHg. However, the dose of amlodipine varied to accommodate individual differences.

A few limitations exist in this investigation. Whether the oral dose of amlodipine level had an effect on the combined folic acid and pravastatin treatment is not clear. Further studies are needed to determine the effects of other drugs taken by elderly patients with hypertension and lacunar infarction on folic acid treatments. We were also limited by the small sample size and short observation time. The exact mechanism of the effects and theoretical verification will need to be further confirmed by a large sample and randomized controlled study.

## Author contributions

**Conceptualization:** Xingpeng Bu.

**Data curation:** Chunxia Li, Xingpeng Bu.

**Formal analysis:** Chunxia Li.

**Funding acquisition:** Yaru Liu.

**Investigation:** Chunxia Li.

**Methodology:** Chunxia Li, Xingpeng Bu.

**Resources:** Xingpeng Bu, Yaru Liu.

**Supervision:** Yaru Liu.

**Validation:** Xingpeng Bu.

**Visualization:** Yaru Liu.

**Writing – original draft:** Chunxia Li, Xingpeng Bu.

**Writing – review & editing:** Chunxia Li.
